# Salivary Biomarker Profiles and Chronic Fatigue among Nurses Working Rotation Shifts: An Exploratory Pilot Study

**DOI:** 10.3390/healthcare10081416

**Published:** 2022-07-28

**Authors:** Shinya Yamaguchi, Kazuhiro Watanabe, Naotaka Sugimura, Inaho Shishido, Issei Konya, Tomoko Fujita, Yuichi Yoshimitsu, Shintaro Kato, Yoichi M. Ito, Rika Yano

**Affiliations:** 1 Graduate School of Health Sciences, Hokkaido University, Sapporo 060-0812, Japan; s_yamaguchi@eis.hokudai.ac.jp (S.Y.); kazu-watanabe@eis.hokudai.ac.jp (K.W.); naotaka-s@eis.hokudai.ac.jp (N.S.); ik-0v0-ik628@eis.hokudai.ac.jp (I.K.); 2 Faculty of Health Sciences, Hokkaido University, Sapporo 060-0812, Japan; inaho_s@hs.hokudai.ac.jp; 3 Research Fellow of Japan Society for the Promotion of Science, Tokyo 102-0083, Japan; 4 NEC Solution Innovators, Ltd., Tokyo 136-8627, Japan; tom-fujita@nec.com (T.F.); yoshimitsu-y@nec.com (Y.Y.); katou-s-mxn@nec.com (S.K.); 5 Biostatics Division, Clinical Research and Medical Innovation Center, Hokkaido University Hospital, Sapporo 060-8648, Japan; ito-ym@med.hokudai.ac.jp

**Keywords:** nurses, fatigue, saliva, biomarkers, shift work schedule

## Abstract

Although nurses’ fatigue affects their well-being and patient safety, no effective objective measurements exist. We explored the profiles of salivary biomarkers associated with nurses’ chronic fatigue across several shifts. This longitudinal study involved 45 shiftwork nurses and collected their saliva samples before two night and two day shifts for a month. Chronic fatigue was measured using the Cumulative Fatigue Symptom Index before the first night shift. Biomarker profiles were analyzed using hierarchical cluster analysis, and chronic fatigue levels were compared between the profiles. Cortisol profiles were classified into high- and low-level groups across two day shifts; the low-level group presented significantly higher irritability and unwillingness to work. Secretory immunoglobulin A (s-IgA) profiles across the four shifts were classified into high- and low-level groups; the high-level group had significantly higher depressive feelings, decreased vitality, irritability, and unwillingness to work. Cortisol (two day shifts) and s-IgA (four shifts) profiles were combined, and (i) cortisol low-level and s-IgA high-level and (ii) cortisol high-level and s-IgA low-level groups were identified. The former group had significantly higher chronic fatigue sign and irritability than the latter group. The profiles of salivary cortisol and s-IgA across several shifts were associated with nurses’ chronic fatigue.

## 1. Introduction

Shiftwork nurses are at an increased risk of chronic fatigue, which occurs as a consequence of work-related stress from excessive work demands and insufficient rest [1,2]. In nursing work, a heavy workload, long working hours including mandatory overtime, and other stressful situations are common [3,4]. Moreover, night shifts and short rest periods between shifts included in shift work can compromise sleep quality and impede recovery from fatigue [5,6], thereby significantly accumulating fatigue and progressing to chronic fatigue [1,7]. Previous studies have reported that nurses’ chronic fatigue is associated with an individual’s mental health problems [8], burnout, turnover intentions [9,10], a decrease in work performance [11,12,13], and medical error [5,14]. Therefore, managing and preventing chronic fatigue for nurses, through screening and early intervention based on effective indicators, is necessary.

As the symptoms of any fatigue are inherently subjective and psychological in nature and because it is necessary to recognize the phenomenon [15], fatigue among nurses is generally measured using a self-administered questionnaire [16]. However, since the questionnaires are based on an individual’s self-awareness, they may not be sufficiently accurate owing to a lack of objectivity. Several biomarkers, including hormones, immunoglobulins, and enzymes, are associated with work-related stress, which can cause fatigue [17]. Blood and urine also contain these biomarkers, but saliva, which can be collected easily and noninvasively, is more useful in a work setting [18]. The choice of these biomarkers to examine physiological responses to work-related stress and fatigue depends on the type of stress investigated, with the hypothalamic–pituitary–adrenal axis and immune function being considered relevant for chronic conditions [17,18,19].

Cortisol, a glucocorticoid hormone secreted by the adrenal cortex, is regulated by negative feedback from the hypothalamus and pituitary gland [19,20,21]. Previous studies have suggested associations between salivary cortisol and fatigue among community-dwelling adults [20], depression [21,22,23], anxiety [24], and chronic fatigue syndrome [25,26,27]. Oxytocin is a neuropeptide associated with social behavior and stress [28,29], it was reported that oxytocin secreted in association with cortisol in experimental studies [30]. A previous study on police officers reported that a post-traumatic stress disorder patient group had significantly lower salivary oxytocin levels than the control group [31]. Secretory immunoglobulin A (s-IgA), the primary component of mucosal antibodies, is an indicator of the activity of the immune system. Chronic stress activates the immune system and inflammatory response by increasing noradrenaline release and pro-inflammatory cytokines production; this is supported by previous studies [32,33] using salivary s-IgA. Conversely, other studies [34,35] have reported that chronic stress is also associated with low secretion of salivary s-IgA.

Although the association between these biomarkers and chronic fatigue in nurses is unclear, several studies have shown an association with nurses’ stress in the context of work. A cross-sectional study [36] compared work-related stress and salivary cortisol levels between emergency department nurses and general ward nurses. The work-related stress of emergency department nurses was significantly higher than that of general ward nurses, whereas the level of salivary cortisol in emergency department nurses (before the day shift) was significantly lower. This result suggested a negative correlation between salivary cortisol levels and work-related stress. In addition, in a study of female healthcare workers engaged in shift work [37], a significant association has been found between night shift and a longer shift duration (more than 8 h) and morning salivary cortisol levels. These findings suggest that cortisol levels, especially in the morning, are associated with work-related stress and chronic fatigue in nurses.

Data on the association between salivary s-IgA and chronic stress in nurses remain inconsistent. Fujimaru et al. [38] and Yang [39] reported a negative correlation between the levels of salivary s-IgA and work-related stress levels, comparing emergency department nurses with general ward nurses and neonatal intensive care unit nurses with general ward nurses. However, longitudinal studies [32,33] with repeated measures of salivary s-IgA showed that higher job stress levels were associated with higher salivary s-IgA levels.

Given the association between work-related stress and chronic fatigue, previous findings are useful in exploring associations between salivary biomarkers and chronic fatigue in nurses. However, salivary biomarker levels can easily change due to factors such as sampling time, lifestyle, and psychological conditions [17,19]. Therefore, to investigate the association between salivary biomarkers and nurses’ fatigue, a study that measures salivary biomarkers over a period is warranted [33,37]. Additionally, considering the obvious diurnal variation in salivary biomarkers and the effects of any work conditions, collecting saliva before each work shift is necessary.

Therefore, the purpose of this pilot study is to explore the possibility of salivary cortisol, s-IgA, and oxytocin as objective indicators of chronic fatigue in nurses. We identified the profiles of salivary cortisol, oxytocin, and s-IgA among nurses across several shifts, and explored the associations between salivary biomarker profiles and nurses’ fatigue levels.

## 2. Materials and Methods

### 2.1. Study Design and Participants

**Figure 1:**
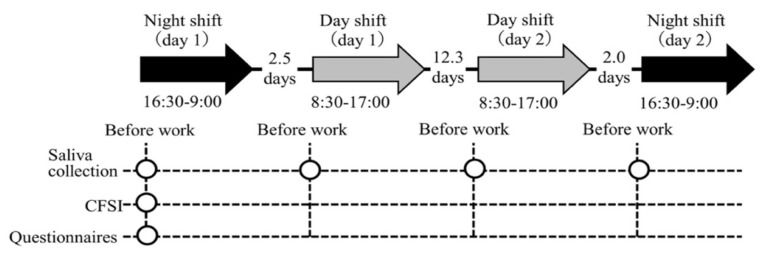
Illustration of the study procedure. Abbreviations: CFSI, Cumulative Fatigue Symptom Index. Note: The arrows indicate the shifts investigated. The intervals between each shift (average number of days) are indicated between arrows. The circles indicate each measurement point.

In this longitudinal study with repeated measures of salivary biomarkers, we investigated each participant in two day and two night shifts for a month. The study protocol is shown in Figure 1. The hospital applied a two-shift system consisting of a day shift (from 08:30 to 17:00) and a night shift (from 16:30 to 09:00); this shift schedule applies to 41.4% of hospital nurses in Japan [40]. Commonly, a nurse’s roster is created by the unit manager on a monthly basis, for which an average of four night shifts are included in the two-shift system. Thus, we identified the periods in a month in which nurses had four night shifts and selected the first and fourth night shifts and the two day shifts in between these night shifts to measure the salivary biomarkers. Day shifts were selected only if the previous day was a day off. Thus, the follow-up period for each participant was approximately one month, which was appropriate for investigating the association between chronic fatigue and the salivary biomarker profiles across several shifts. The order of the four shifts was night shift (day 1), day shift (day 1), day shift (day 2), and night shift (day 2) for all participants. This study was conducted between July and September 2019.

The participants were female nurses who worked in the two-shift system in the six wards in a general hospital with >200 beds in northern Japan. Only female nurses were included, given the sex-based differences in salivary biomarker levels. In addition, considering the modulation of the hormonal balance, only nurses in their 20 s to 30 s were included. The exclusion criteria were as follows: (1) disease with symptoms of fatigue (e.g., anemia); (2) less than 1 year of experience; (3) regular use of sleeping pills; and (4) pregnancy. Because this pilot study is an exploratory study, sample size calculations could not be performed. Participants were informed about the study aims, methods, and anonymity-related procedures were explained verbally and in writing. In addition, participants provided written and verbal informed consent for publication before participating in this study. This study was conducted in accordance with the Declaration of Helsinki and approved by the Institutional Review Board of the university to which the authors are affiliated and the participating facility (reference No. 19–1).

### 2.2. Measurements

#### 2.2.1. Chronic Fatigue

The Cumulative Fatigue Symptom Index (CFSI) [41] was used to measure the participants’ fatigue. The CFSI consists of 81 items concerning physical and mental complaints and daily life conditions of workers, and participants answer all questions with two choices (yes/no). The items are divided into eight categories, and these categories are further subdivided into three aspects as follows: (1) the physical aspect (general fatigue, chronic fatigue sign, and physical disorders); (2) the mental aspect (depressive feelings, anxiety, and decreased vitality); (3) the social aspect (irritability and unwillingness to work). The complaint rate (%) for each category is calculated as follows:

$$\begin{array}{cc} & \mathrm{Complaint}\;\mathrm{rate}\;(\%)\;=\;(\mathrm{number}\;\mathrm{of}\;\mathrm{positive}\;\mathrm{items})\;÷\;(\mathrm{number}\;\mathrm{of}\;\mathrm{items}\;\times \;\mathrm{number}\;\mathrm{of}\;\\ & \mathrm{participants})\;\times \;100 \end{array}$$

The CFSI can assess symptoms or discomfort that are felt recently or over time, and a previous study [42] confirmed that scale scores do not vary from week to week or shift to shift. Therefore, the CFSI was not measured at the time of each shift, and participants were asked to complete a questionnaire at the start of the first night shift. In this way, it was assumed that a participant’s chronic fatigue during the study period could be assessed.

#### 2.2.2. Saliva Sampling and Analysis

Saliva was collected from the participants before the start of each shift. Sarisoft (Funakoshi, Tokyo, Japan; catalog code: 51.1534.901S), an integrated sponge and tube, was used. For standardization, participants were asked to refrain from eating, smoking, drinking, and brushing their teeth 1 h before saliva collection [16,18]. In addition, considering the diurnal change in each biomarker level, the saliva collection time for all shifts was unified before work. The collection procedure was as follows. First, the sponge was attached to the Sarisoft in the oral cavity and allowed to remain for 3 min, with the participant in a sitting position. Then, the sponge was removed from the oral cavity, stored in a collection tube, and sealed. After collection, the sample was moved to a dedicated freezer and stored frozen at −20 °C or lower. Since the saliva collection period was in the summer, a portable cooler was used when moving the specimens. The frozen saliva samples were naturally thawed at room temperature (RT) and then centrifuged (3000× *g*, 2 min, RT), and the saliva at the bottom of the Sarisoft was collected in a 1.5 mL tube. Each biomarker in saliva was measured using the kit described as follows according to the manual attached to the kit.

The salivary cortisol concentration was measured using the Cortisol (Saliva) EIA kit (Yanaihara Laboratory, Shizuoka, Japan; catalog number: YK241). In total, 50 μL of the prepared saliva and 150 μL of the labeled antibody solution were added and shaken for the reaction (210–220 rpm, 1 h, RT). The reaction solution in the well of the microplate was discarded; after washing seven times with the washing solution, 100 μL of the enzyme substrate solution was added to the well, and the reaction was shaken (210–220 rpm, 30 min, RT, shading light). After adding 100 μL of the enzyme reaction terminator, the absorbance at 450 nm was measured with an Infinite M1000Pro microplate reader (TECAN Japan, Kanagawa, Japan).

An ELISA Kit for Secretory Immunoglobulin A (Cloud-clone, Houston, TX, USA; catalog number: SEA641HU) was used to determine the s-IgA concentration. Saliva samples (100 μL) were added to the wells of the microplate, and the reaction was allowed to stand (1 h, 37 °C). The solution was removed from the well, 100 μL of reagent A was added, and the reaction was allowed to stand (1 h, 37 °C). After removing reagent A and washing three times with a washing solution, 100 μL of reagent B was added and allowed to react (30 min, 37 °C). Reagent B was removed, the mixture was washed five times with a washing solution, and then, the substrate was added to the wells and the mixture was exposed to light and allowed to stand (15 min, 37 °C). After adding 50 μL of the enzyme reaction terminator, the absorbance at 450 nm was measured.

The oxytocin concentration was measured using an Oxytocin EIA kit (Cayman, MI, USA; item number: 500440). Next, 100 μL of the prepared saliva sample was added to the wells of the microplate, 50 μL of oxytocin AChE Tracer and 50 μL of the antibody solution were added, and the reaction was allowed to stand (18 h, 4 °C). Then, 200 μL of the prepared Ellman’s reagent was added, and after blocking light and shaking the reaction (500 rpm, 90 min, RT), the absorbance at 412 nm was measured.

The concentration of the biomarkers in each saliva sample was calculated from the standard curve prepared from the four-parameter logistic regression curve and the absorbance value. The absorbance of each saliva sample was measured based on two wells, and the average value was used to calculate the biomarker concentration. According to the information in the respective manuals, the standard ranges for the determination of cortisol, oxytocin, and s-IgA were 0.012–3.000 µg/dL, 5.9–750.0 pg/mL, and 0.027–20.000 ng/mL with a sensitivity of 0.046 µg/dL, 20.0 pg/mL, and <0.010 ng/mL at the 95% confidence limit, respectively. The intra-assay coefficients of variation for cortisol, oxytocin, and s-IgA were <5.0, <16.0, and <10.0% with inter-assay coefficients of variation of <6.0, <15.0, and <12.0%, respectively.

#### 2.2.3. Demographic Data

An original demographic self-administered questionnaire was distributed to the participants, and the following data were collected: age, height, weight, years of experience, years in the current work setting, marital status, and having children. Information about the disease under treatment was based on the participant’s self-report. Additionally, commute time (one way) and overtime work the previous month (>10 h, 10–19 h, 20–29 h, and ≥30 h) were assessed.

### 2.3. Statistical Analysis

All statistical analyses were performed using the JMP Pro software, ver.16.0 (SAS Institute Inc., Cary, NC, USA), with the significance level set to 5%. Unless otherwise specified, each variable was expressed using the median (interquartile range, IQR) for continuous variables and the frequency (%) for categorical variables. The data for salivary cortisol, oxytocin, and s-IgA at each shift were skewed; therefore, a logarithmic transformation was performed on these data. For ease of interpretation, the results show the untransformed data.

Spearman’s correlation analysis was performed to examine the association between CFSI and cortisol, s-IgA, and oxytocin levels at each time point. There was a weak positive correlation between s-IgA levels at the four shifts and unwillingness to work (r = 0.32–0.35); however, no consistent and sufficient correlation was found between biomarker levels at a single point and the other categories of the CFSI. Moreover, the CFSI is a scale designed to measure the fatigue of a group rather than an individual. Thus, to analyze the association with the salivary biomarkers, it is necessary to identify groups for each salivary biomarker. Hierarchical cluster analysis (HCA) using Ward’s method was conducted to classify the profiles of each biomarker. In this analysis, participants with a missing value for any of the measurement points were excluded (i.e., complete-case analysis), and the classification was made sequentially from participants or groups with similar levels of each biomarker across four shifts. Additionally, as previous studies [21,26] suggested that morning cortisol levels are associated with chronic fatigue syndrome and depression, we also conducted an HCA of cortisol based on the data from two day shifts. Further, an HCA based on combining the six variables of cortisol and s-IgA, that is, cortisol (across the two day shifts) and s-IgA (across the four shifts), was performed. This resulted in a group in which the profile of both cortisol and s-IgA measured across several shifts was considered.

The Mann–Whitney U test was conducted to compare the complaint rate of the CFSI (dependent variable) among the profiles of each salivary biomarker (independent variable). In this exploratory pilot study, no adjustment for multiplicity of tests was performed since power analysis was not considered a priori. Further, no adjustment for covariates was made owing to the small sample size. The effect size (r) was reported by calculating $r\;=\left|z\right|\;/\;\sqrt{n}$ where z is the Z statistic and *n* indicates the total sample size, and we adopted the criteria of r = 0.10, 0.30, and 0.50 to indicate small, medium, and large effect sizes, respectively. For comparison of participant characteristics in each biomarker profiles, the Mann–Whitney U test for continuous variables and the chi-squared test or Fisher’s exact test for categorical variables were performed.

## 3. Results

### 3.1. Participants’ Characteristics

Forty-seven participants were recruited for this study, of which two were excluded from the analysis, including one who declined and one who reported anemia, a disease with fatigue as a symptom. Table 1 summarizes the participants’ characteristics. Of the participants, 24 (53.3%) were in their 20 s and 21 (46.7%) were in their 30 s. A total of 30 (66.7%) participants worked in the medical wards and 15 (33.3%) in the surgical wards.

### 3.2. Salivary Biomarkers

In total, 180 saliva samples were collected from the 45 participants (Supplementary Figure S1 and Table S1). Concentration could not be determined for samples with insufficient saliva volume and those below the detection limit were excluded from the analysis. Data that were outliers (high values) were excluded from the analysis because they were not classified appropriately in the HCA.

### 3.3. Chronic Fatigue

Among the categories of the CFSI, the chronic fatigue sign (75.0%) was the highest, followed by decreased vitality (44.4%) and general fatigue (40.0%). Further, the categories with low complaint rates were irritability (14.3%) and unwillingness to work (23.1%), both of which belong to the social aspect.

### 3.4. Profiles of Salivary Biomarkers and Chronic Fatigue

**Figure 2:**
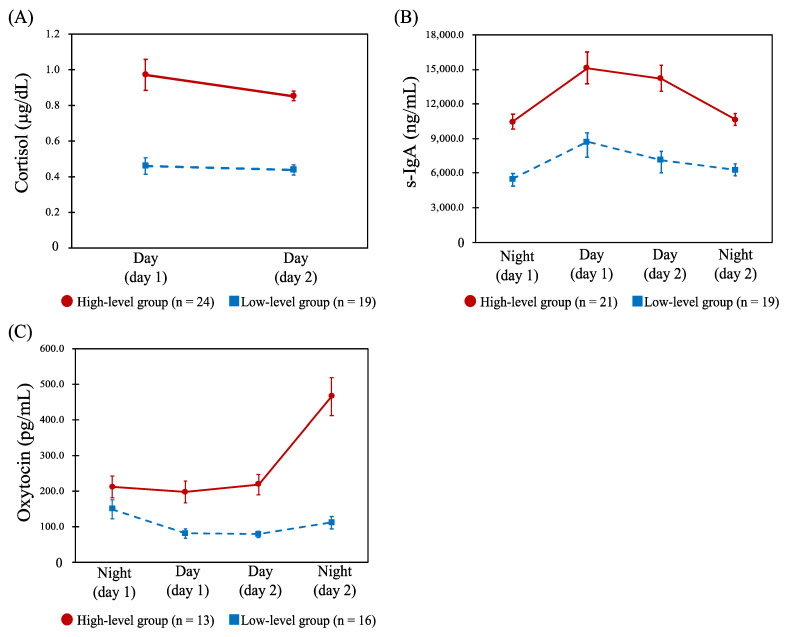
Profiles of the salivary cortisol, secretory immunoglobulin A (s-IgA), and oxytocin. Abbreviations: Day, day shift; Night, night shift; s-IgA, secretory immunoglobulin A. Note: Data are shown as the mean (error bar, standard error of measurement) of each biomarker concentration. (**A**): Dynamics of the salivary cortisol concentration (μg/dL) between day shifts in the high-level (*n* = 24) and low-level groups (*n* = 19). (**B**): Dynamics of the s-IgA concentration (ng/mL) between the four shifts in the high- (*n* = 21) and low-level groups (*n* = 19). (**C**): Dynamics of the salivary oxytocin concentration (pg/mL) between the four shifts in the high- (*n* = 13) and low-level groups (*n* = 16).

For cortisol, the profiles across the four shifts were classified into high- (*n* = 11) and low-level groups (*n* = 29). Table 2 shows the results of comparing the CFSI complaint rates (%) between these profiles, and there were no statistically significant differences for any of the categories. Further, HCA was conducted on the two day shifts’ data only, and similarly the high- (*n* = 24) and low-level groups (*n* = 19) were identified (Figure 2A). There were significant differences in complaint rates of unwillingness to work (*p* = 0.033) and irritability (*p* = 0.026) between the two groups, both of which were significantly higher in the low-level group (Table 2). Similarly, the profiles of s-IgA across the four shifts were classified into high- (*n* = 21) and low-level groups (*n* = 19) (Figure 2B). Comparing the CFSI, the high-level group had a significantly higher complaint rate than the low-level group in the following categories belonging to the mental or social aspects, as shown in Table 2: depressive feelings (*p* = 0.034), decreased vitality (*p* = 0.026), unwillingness to work (*p* = 0.005), and irritability (*p* = 0.041). The profiles of oxytocin for the four shifts were also classified into high- (*n* = 13) and low-level groups (*n* = 16) (Figure 2C). No statistically significant difference was observed when comparing the CFSI between the two groups.

### 3.5. Comparison of Chronic Fatigue between Combined Profiles of Cortisol and s-IgA

**Figure 3:**
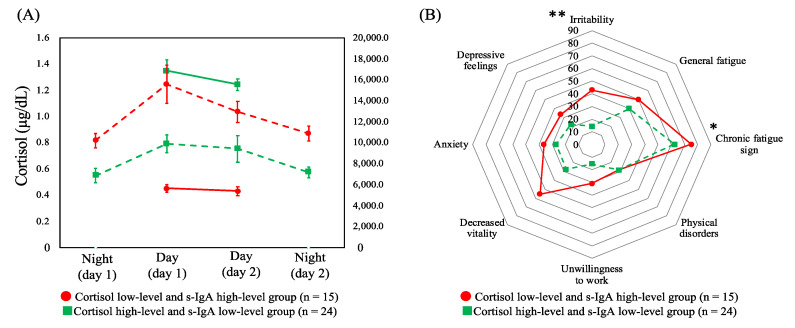
(**A**) Dynamics of cortisol levels across two day shifts (solid line) and secretory immunoglobulin A levels (dashed line) across four shifts of profiles of both combined biomarkers with means and standard errors. (**B**) Comparison of the median complaint rate (%) of the Cumulative Fatigue Symptom Index between profiles. Abbreviations: s-IgA = secretory immunoglobulin A. Note: * *p* < 0.050, ** *p* < 0.010.

Based on the aforementioned results, HCA was performed by combining cortisol (across the two day shifts) and s-IgA (across four shifts), the two salivary biomarkers with significant differences in terms of the CFSI complaint rates. HCA was performed on the six variables, which were identified and comprised the following: (i) low levels of cortisol in two day shifts and high levels of s-IgA in the four shifts (cortisol low-level and s-IgA high-level group, *n* = 15) and (ii) high-levels of cortisol and low-levels of s-IgA (cortisol high-level and s-IgA low-level group, *n* = 24). Figure 3A shows the dynamics of cortisol and s-IgA levels of these two groups. As shown in Figure 3B, the complaint rates of the CFSI were significantly higher in the cortisol low-level and s-IgA high-level group than in the cortisol high-level and s-IgA low-level group in the following categories belonging to the physical or social aspect: chronic fatigue sign (*p* = 0.041) and irritability (*p* = 0.004).

### 3.6. Association between Salivary Biomarkers and Participant Characteristics

Variables related to participant characteristics were compared between the groups of each biomarker. No statistically significant differences were observed for any of the variables in the salivary cortisol profiles, s-IgA profiles, and profiles of two biomarkers combined (Supplementary Tables S2–S5).

## 4. Discussions

In this study, we examined the association between the profiles of salivary biomarkers in several shifts and nurses’ chronic fatigue. The profiles of each salivary biomarker were classified via HCA, and the complaint rates of the CFSI were compared among the classified groups, demonstrating that cortisol was significantly associated with two items attributable to the social aspects of fatigue and that s-IgA was significantly associated with four items attributable to the mental or social aspects of fatigue. Interestingly, combining cortisol (the two day shifts) and s-IgA (the four shifts) showed a significant association with chronic fatigue sign (physical aspect), not identified for each salivary biomarker. Based on the close association between multiple stress systems [17,43], for a comprehensive assessment of nurses’ fatigue, which has a multidimensional structure [2], cortisol, s-IgA, and the combination of these biomarkers might be effective indicators. In addition, the effects of nurses’ fatigue vary according to physical or mental aspects [8,13]. Thus, our findings imply the need to measure salivary cortisol and s-IgA in combination to effectively address fatigue risk.

Regarding cortisol in two day shifts, the low-level group had higher levels of social fatigue. Previous studies [21,22,25,26,36,37] have reported that salivary cortisol levels, especially measured in the morning, are associated with depression, chronic fatigue syndrome, and worker stress, which is consistent with our results. As cortisol regulates body and brain activity during arousal, including energy production, metabolism, and mood [44,45], nurses who belong to the cortisol low-level group may not function optimally. This suggests that those belonging to the cortisol low-level group had a higher level of chronic fatigue.

We found that in the profile of s-IgA, fatigue was higher in the high-level group than in the low-level group. The difference between the two groups was observed in the s-IgA levels. With respect to this, different results have been reported among nurses. Cross-sectional studies [38,39] have shown that salivary s-IgA levels are negatively associated with work-related stress. However, a longitudinal study identified that the higher the job stress level, the higher the s-IgA levels [32,33]. Our result was consistent with the latter finding, suggesting that among nurses with higher fatigue, this would be explained by the change in immune function, such as the Th1-to-Th2 model shift, which causes Th2 cytokines to activate humoral immunity [33].

Despite the seriousness of nurses’ chronic fatigue, no effective measurement method has been established. As fatigue is commonly described as subjective, questionnaires have been primarily used for its assessment. However, considering the possibility that stress and fatigue can cause various physiological responses, nurses’ chronic fatigue could be understood more accurately by incorporating an assessment of other aspects [15]. Therefore, to sufficiently understand nurses’ chronic fatigue, establishing an assessment method combining self-administered questionnaires and objective indicators is necessary. The present study suggests that salivary cortisol and s-IgA are potential biomarkers associated with chronic fatigue in nurses. Further investigations on the association between these profiles and burnout syndrome, which is caused by chronic fatigue and can lead to nurse turnover [46,47], will provide deeper insight into the utility of salivary biomarkers in nursing management. Additionally, further studies with larger sample sizes are also warranted for more data on the association between chronic fatigue in nurses and salivary cortisol and s-IgA and to confirm the prediction accuracy and cut-off values.

This study has some limitations. The first is external validity. This study was conducted at a single hospital in a specific area in Japan and was limited to female nurses in the age group of 20–30 years with a relatively small sample. In addition, we did not set a control population of women with similar demographics for comparison. Therefore, it is not clear whether our findings are specific to shiftwork nurses or whether they are transferable to other populations. Second, in this exploratory pilot study, a power analysis was not performed. Therefore, in each statistical test, strict multiplicity adjustment was not performed; thus, the problem of false positives must be considered. Third, there were a relatively high number of samples in which the oxytocin concentration could not be determined owing to insufficient volume of saliva. Oxytocin, a neuropeptide, was investigated for its ability to buffer various stresses. Our analysis found a moderate effect size for the difference in the CFSI complaint rates in the oxytocin group (Table 2). A study with larger sample size and saliva collection time is warranted to verify the association between salivary oxytocin and nurses’ chronic fatigue. Finally, we could not consider the effects of other variables, such as work conditions, sleep conditions, and diet conditions on the day before sampling, as well as internal factors (e.g., chronotype) which could have influenced the concentration of the salivary biomarkers.

## 5. Conclusions

Our findings indicated the following possibilities: (i) the profiles of salivary cortisol and s-IgA based on differences in levels across several shifts could be associated with nurses’ chronic fatigue; (ii) to provide a comprehensive fatigue assessment that includes physical fatigue, measuring cortisol and s-IgA not only separately but also in combination could be effective.

## Figures and Tables

**Table 1:** Participants’ characteristics (*n* = 45).

	Median (IQR)	Number (%)
Age, years	29.0 (26.0, 32.0)	
BMI, kg/m^2^	21.1 (19.3, 22.2)	
Years as nurse, years	7.0 (3.0, 9.0)	
Years in current work setting, years	3.0 (2.0, 4.5)	
Marital status		
Married		8 (17.8)
Single		37 (82.2)
Have children		
Yes		4 (8.9)
No		41 (91.1)
Commute time (one way), min ^a^	30.0 (22.5, 38.8)	
Overtime hours (last month)		
<10		30 (66.7)
10–19		13 (28.9)
20–29		2 (4.4)
≥30		0 (0)

Abbreviations: BMI = body mass index; IQR = interquartile range. Note: ^a^ *n* = 44.

**Table 2:** Comparison of chronic fatigue by profiles of each salivary biomarker.

	Physical Aspect			Mental Aspect			Social Aspect	
	General Fatigue	Chronic Fatigue Sign	Physical Disorder	Depressive Feelings	Anxiety	Decreased Vitality	Unwillingness to Work	Irritability
Cortisol (four shifts)								
HL group (*n* = 11)	50.0 (30.0, 70.0)	75.0 (37.5, 87.5)	14.3 (14.3, 57.1)	44.4 (22.2, 66.7)	36.4 (9.1, 54.6)	66.7 (22.2, 66.7)	15.4 (15.4, 69.2)	0 (0, 42.9)
LL group (*n* = 29)	40.0 (30.0, 60.0)	75.0 (43.8, 75.0)	28.6 (14.3, 57.1)	33.33 (16.7, 55.6)	27.3 (9.1, 50.0)	55.6 (22.2, 66.7)	23.1 (11.5, 38.5)	14.3 (0, 42.9)
*p*-Value	0.187	0.579	0.687	0.445	0.783	0.976	0.561	0.235
Effect size (r)	0.21	0.09	0.06	0.12	0.04	0	0.09	0.19
Cortisol (day shifts)								
HL group (*n* = 24)	40.0 (30.0, 60.0)	62.50 (28.1, 75.0)	28.57 (14.3, 53.6)	27.78 (11.1, 63.9)	27.27 (2.3, 52.3)	27.8 (11.1, 66.7)	15.4 (7.7, 34.6)	14.3 (0, 25.0)
LL group (*n* = 19)	50.0 (30.0, 60.0)	75.00 (62.5, 87.5)	28.6 (28.6, 57.1)	33.3 (33.3, 55.6)	27.3 (18.2, 45.5)	55.6 (33.3, 77.8)	30.8 (15.4, 61.5)	28.6 (14.3, 42.9)
*p*-Value	0.911	0.062	0.194	0.251	0.323	0.058	0.033	0.026
Effect size (r)	0.02	0.28	0.20	0.17	0.15	0.29	0.33	0.34
Oxytocin (four shifts)								
HL group (*n* = 13)	50.0 (35.0, 70.0)	75.0 (62.5, 87.5)	57.1 (14.3, 64.3)	44.4 (22.2, 66.7)	27.3 (22.7, 50.0)	66.7 (22.2, 72.2)	23.1 (15.4, 61.5)	14.3 (0, 57.1)
LL group (*n* = 16)	35.0 (30.0, 50.0)	62.5 (28.1, 75.0)	28.6 (3.6, 39.3)	27.8 (11.1, 44.4)	31.8 (9.1, 54.6)	33.3 (11.1, 55.6)	19.2 (9.6, 36.5)	14.3 (0, 42.9)
*p*-Value	0.096	0.099	0.112	0.138	0.842	0.102	0.672	0.602
Effect size (r)	0.31	0.31	0.30	0.27	0.04	0.30	0.08	0.10
s-IgA (four shifts)								
HL group (*n* = 21)	40.0 (30.0, 60.0)	75.0 (56.2, 87.5)	28.6 (14.3, 57.1)	44.4 (33.3, 72.2)	36.4 (22.7, 50.0)	66.7 (38.9, 77.8)	38.5 (19.2, 69.2)	28.6 (14.3, 50.0)
LL group (*n* = 19)	40.0 (30.0, 60.0)	62.5 (37.5, 75.0)	28.6 (14.3, 57.1)	22.2 (11.1, 55.6)	27.3 (0, 54.6)	33.3 (11.1, 66.7)	15.4 (7.7, 23.1)	14.3 (0, 28.6)
*p*-Value	0.848	0.215	0.857	0.034	0.235	0.026	0.005	0.041
Effect size (r)	0.03	0.20	0.03	0.33	0.19	0.35	0.44	0.32

Abbreviations: s-IgA, secretory immunoglobulin A; HL, high-level; LL, low-level. Note: Values are median (interquartile range). The Mann–Whitney U test was used to compare the complaint rates of the cumulative fatigue symptom index of each group. The effect size (r) was reported by calculating $r\;=\left|z\right|\;/\;\sqrt{n}$ using the z statistic.

## Data Availability

Not applicable.

## Supplementary Materials

The following supporting information can be downloaded at: https://www.mdpi.com/article/10.3390/healthcare10081416/s1, Figure S1: Flow diagram for the participant and salivary biomarker data; Table S1: Salivary cortisol, s-IgA, and oxytocin concentrations for day and night shifts; Table S2: Comparison of participant characteristics based on profiles of salivary cortisol (across two day shifts); Table S3: Comparison of participant characteristics based on profiles of salivary secretory immunoglobulin A(across four shifts); Table S4: Comparison of participant characteristics based on profiles of salivary oxytocin (across four shifts); Table S5: Comparison of participant characteristics based on profiles of combined cortisol and secretory immunoglobulin A.
