# Supplementary material for: Salivary Biomarker Profiles and Chronic Fatigue among Nurses Working Rotation Shifts: An Exploratory Pilot Study
Source: Healthcare (Basel). 2022 Jul 28;10(8):1416. doi: 10.3390/healthcare10081416 (PMC9407778; doi:10.3390/healthcare10081416)
Supplement: Supplementary file 1 [file healthcare-10-01416-s001.zip › Supplementary File/Helathcare_Supplementary Figure S1.pdf]

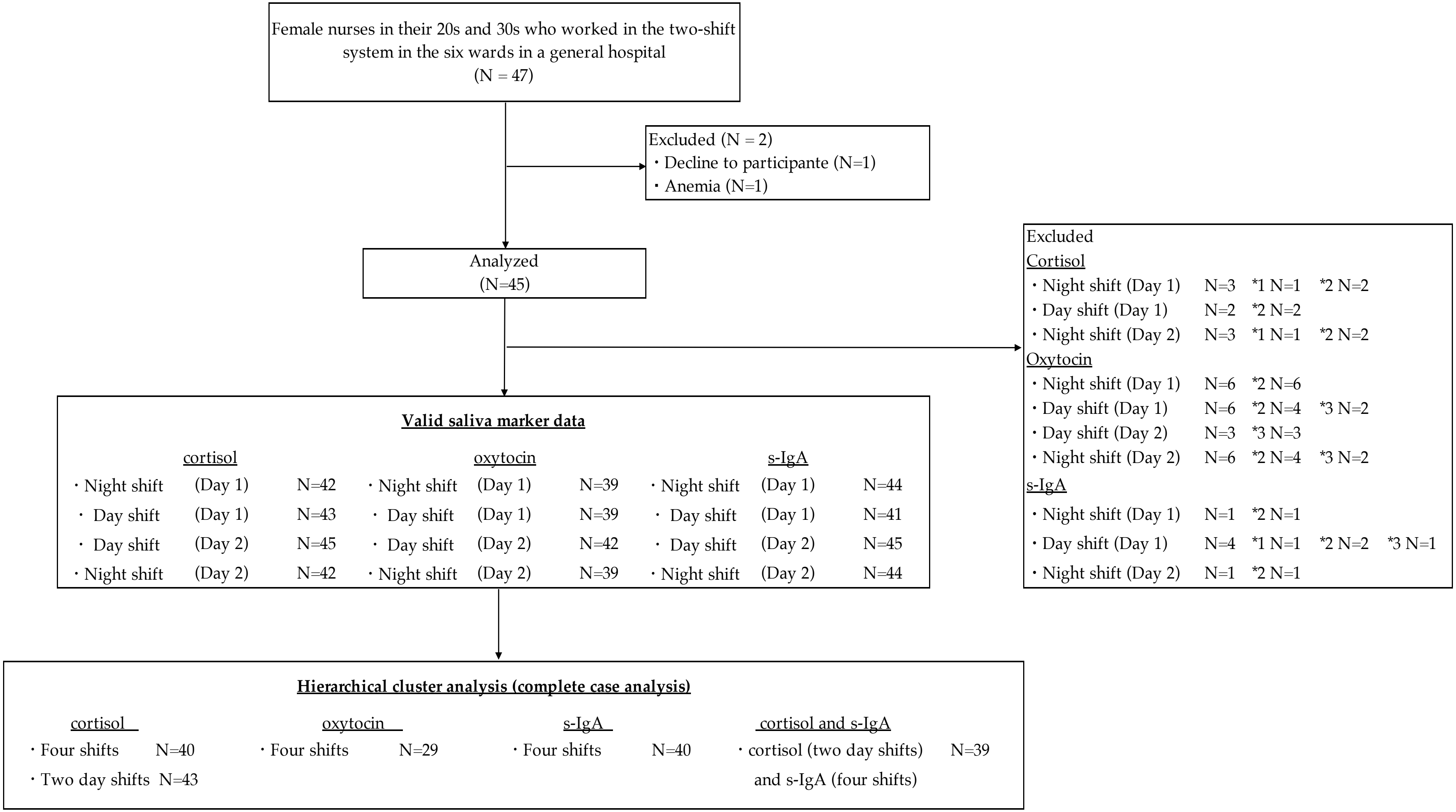

Supplementary Figure S1.
Flow diagram for the participants and salivary biomarkers data
Abbreviation: s-IgA, secretory immunoglobulin A.
*1 Outlier (high value) data not classified in hierarchical cluster analysis.
*2 Data where concentration could not be detected due to lack of saliva volume in the sample.
*3 Data for which concentration was below the limit of detection.
