# Supplementary material for: Salivary Biomarker Profiles and Chronic Fatigue among Nurses Working Rotation Shifts: An Exploratory Pilot Study
Source: Healthcare (Basel). 2022 Jul 28;10(8):1416. doi: 10.3390/healthcare10081416 (PMC9407778; doi:10.3390/healthcare10081416)
Supplement: Supplementary file 1 [file healthcare-10-01416-s001.zip › Supplementary File/Healthcare_Supplementary Table S3.pdf]

**Supplementary Table S3.**

Comparison of participant characteristics based on profiles of salivary secretory immunoglobulin A(across four shifts).

| | s-IgA high-level group (n=21) | s-IgA low-level group (n=19) | *p*-Value |
|---|---|---|---|
| Age, years | 28.0 (24.5, 32.0) | 29.0 (26.0, 31.0) | 0.78 |
| BMI, kg/m2 | 21.4 (20.0, 23.2) | 21.1 (19.2, 21.9) | 0.11 |
| Years as nurse, years | 7.0 (4.0, 8.5) | 7.0 (4.0, 9.0) | 0.55 |
| Years in current work setting, years | 3.0 (1.0, 4.0) | 3.0 (2.0, 5.0) | 0.30 |
| Marital status | | | |
| Married | 3 (14.3) | 4 (21.0) | 0.69 |
| Single | 18 (85.7) | 15 (79.0) | |
| Having children | | | |
| Yes | 2 (9.5) | 1 (5.3) | 0.99 |
| No | 19 (90.5) | 18 (94.7) | |
| Commute time (one way), min | 30.0 (30.0, 35.0) | 30.0 (20.0, 45.0) | 0.49 |
| Overtime work (last month) | | | |
| < 10 h | 14 (66.7) | 14 (73.7) | 0.86 |
| 10-19 h | 6 (28.6) | 4 (21.0) | |
| 20-29 h | 1 (4.8) | 1 (5.3) | |
| ≧30 h | 0 (0) | 0 (0) | |
| Ward | | | |
| Medical ward | 13 (61.9) | 15 (79.0) | 0.31 |
| Surgical ward | 8 (38.1) | 4 (21.0) | |

***Abbreviations***: BMI, body mass index; s-IgA, secretory immunoglobulin A.

***Note***: Values are median (interquartile range) or the number of participants (%). Differences in continuous variables were assessed using the Mann-Whitney U-test. Differences in categorical variables were assessed using the Chi-squared test or Fisher's exact test.
