# Supplementary material for: Salivary Biomarker Profiles and Chronic Fatigue among Nurses Working Rotation Shifts: An Exploratory Pilot Study
Source: Healthcare (Basel). 2022 Jul 28;10(8):1416. doi: 10.3390/healthcare10081416 (PMC9407778; doi:10.3390/healthcare10081416)
Supplement: Supplementary file 1 [file healthcare-10-01416-s001.zip › Supplementary File/Healthcare_Supplementary Table S2.pdf]

Supplementary Table S2.

Comparison of participant characteristics based on profiles of salivary cortisol (across two day shifts).

	Cortisol high-level group (n=24)	Cortisol low-level group (n=19)	*p*-Value
Age, years	27.5 (24.3, 31.8)	30.0 (28.0, 33.0)	0.17
BMI, kg/m2	20.9 (19.3, 22.7)	21.1 (19.4, 22.2)	0.97
Years as nurse, years	5.5 (2.3, 9.0)	7.0 (3.0, 9.0)	0.40
Years in current work setting, years	2.0 (2.0, 4.8)	3.0 (1.0, 5.0)	0.72
Marital status			
Married	2 (8.3)	5 (26.3)	0.21
Single	22 (91.7)	14 (73.7)	
Having children			
Yes	0 (0)	3 (15.8)	0.08
No	24 (100.0)	16 (84.2)	
Commute time (one way), min	30.0 (30.0, 45.0)	30.0 (20.0, 40.0)	0.65
Overtime work (last month)			
< 10 h	15 (62.5)	13 (68.4)	0.66
10-19 h	7 (29.2)	6 (31.6)	
20-29 h	2 (8.3)	0 (0)	
≧30 h	0 (0)	0 (0)	
Ward			
Medical ward	16 (66.7)	13 (68.4)	0.99
Surgical ward	8 (33.3)	6 (31.6)	

Abbreviations: BMI, body mass index.

Note: Values are median (interquartile range) or the number of participants (%). Differences in continuous variables were assessed using the Mann-Whitney U-test. Differences in categorical variables were assessed using the Chi-squared test or Fisher's exact test.
