# Supplementary material for: Salivary Biomarker Profiles and Chronic Fatigue among Nurses Working Rotation Shifts: An Exploratory Pilot Study
Source: Healthcare (Basel). 2022 Jul 28;10(8):1416. doi: 10.3390/healthcare10081416 (PMC9407778; doi:10.3390/healthcare10081416)
Supplement: Supplementary file 1 [file healthcare-10-01416-s001.zip › Supplementary File/Healthcare_Supplementary Table S1.pdf]

Supplementary Table S1.

Salivary cortisol, s-IgA, and oxytocin concentrations for day and night shifts.

	Day shift		Night shift	
	Day 1	Day 2	Day 1	Day 2
Cortisol	0.64	0.66	0.43	0.39
(μg/dL)	(0.48, 0.88) †1	(0.46, 0.84) †2	(0.35, 0.63) †3	(0.30, 0.59) †3
s-IgA	10649.52	9513.87	8204.48	8445.79
(ng/mL)	(8593.40, 15508.30) †4	(6367.86, 13908.51) †2	(5608.26, 10331.89) †5	(6551.35, 11489.92) †5
Oxytocin	83.81	93.77	180.57	174.15
(pg/mL)	(52.67, 202.99) †6	(70.79, 183.74) †3	(59.93, 271.69) †6	(68.88, 356.07) †6

Abbreviations: s-IgA, secretory immunoglobulin A.

Note: Values are median (interquartile range).

†1 n = 43, †2 n = 45, †3 n = 42, †4 n = 41, †5 n = 44, †6 n = 39
