# Supplementary material for: Salivary Biomarker Profiles and Chronic Fatigue among Nurses Working Rotation Shifts: An Exploratory Pilot Study
Source: Healthcare (Basel). 2022 Jul 28;10(8):1416. doi: 10.3390/healthcare10081416 (PMC9407778; doi:10.3390/healthcare10081416)
Supplement: Supplementary file 1 [file healthcare-10-01416-s001.zip › Supplementary File/Healthcare_Supplementary Table S5.pdf]

Supplementary Table S5.

Comparison of participant characteristics based on profiles of combined cortisol and secretory immunoglobulin A.

	Cortisol low-level and s-IgA high-level group (n=15)	Cortisol high-level and s-IgA low-level group (n=24)	*p*-Value
Age, years	30.0 (26.0, 33.0)	28.0 (24.3, 31.0)	0.43
BMI, kg/m2	21.4 (19.9, 22.3)	21.3 (19.3, 22.7)	0.61
Years as nurse, years	7.0 (3.0, 9.0)	6.5 (2.3, 9.0)	0.83
Years in current work setting, years	3.0 (1.0, 3.0)	2.5 (2.0, 5.0)	0.45
Marital status			
Married	4 (26.7)	3 (12.5)	0.40
Single	11 (73.3)	21 (87.5)	
Having children			
Yes	2 (13.3)	1 (4.2)	0.55
No	13 (86.7)	23 (95.8)	
Commute time (one way), min	30.0 (30.0, 40.0)	30.0 (20.0, 35.0)	0.42
Overtime work (last month)			
< 10 h	10 (66.7)	17 (70.9)	0.53
10-19 h	5 (33.3)	5 (20.8)	
20-29 h	0 (0)	2 (8.3)	
≧30 h	0 (0)	0 (0)	
Ward			
Medical ward	10 (66.7)	17 (70.8)	0.99
Surgical ward	5 (33.3)	7 (29.2)	

Abbreviations: BMI, body mass index; s-IgA, secretory immunoglobulin A.

Note: Values are median (interquartile range) or the number of participants (%). Differences in continuous variables were assessed using the Mann-Whitney U-test. Differences in categorical variables were assessed using the Chi-squared test or Fisher's exact test.
