# Supplementary material for: Salivary Biomarker Profiles and Chronic Fatigue among Nurses Working Rotation Shifts: An Exploratory Pilot Study
Source: Healthcare (Basel). 2022 Jul 28;10(8):1416. doi: 10.3390/healthcare10081416 (PMC9407778; doi:10.3390/healthcare10081416)
Supplement: Supplementary file 1 [file healthcare-10-01416-s001.zip › Supplementary File/Healthcare_Supplementary Table S4.pdf]

Supplementary Table S4.

Comparison of participant characteristics based on profiles of salivary oxytocin (across four shifts).

	Oxytocin high-level group (n=13)	Oxytocin low-level group (n=16)	*p*-Value
Age, years	29.0 (25.5, 30.5)	29.0 (24.3, 33.0)	0.93
BMI, kg/m2	21.0 (19.3, 22.2)	21.7 (19.8, 23.2)	0.26
Years as nurse, years	7.0 (3.5, 10.0)	7.0 (2.3, 9.8)	0.83
Years in current work setting, years	3.0 (2.0, 6.0)	3.0 (1.3, 5.0)	0.58
Marital status			
Married	2 (15.4)	4 (25.0)	0.66
Single	11 (84.6)	12 (75.0)	
Having children			
Yes	1 (7.7)	2 (12.5)	0.99
No	12 (92.3)	14 (87.5)	
Commute time (one way), min	30.0 (25.0, 30.0)	30.0 (20.0, 50.0)	0.25
Overtime work (last month)			
< 10 h	8 (61.5)	11 (68.8)	0.83
10-19 h	5 (38.5)	4 (25.0)	
20-29 h	0 (0)	1 (6.3)	
≧30 h	0 (0)	0 (0)	
Ward			
Medical ward	8 (61.5)	13 (81.3)	0.31
Surgical ward	5 (38.5)	3 (18.7)	

Abbreviations: BMI, body mass index.

Note: Values are median (interquartile range) or the number of participants (%). Differences in continuous variables were assessed using the Mann-Whitney U-test. Differences in categorical variables were assessed using the Chi-squared test or Fisher's exact test.
